# Qualitative Inquiry into Premarital Sexual Behaviours and Contraceptive Use among Multiethnic Young Women: Implications for Education and Future Research

**DOI:** 10.1371/journal.pone.0051745

**Published:** 2012-12-14

**Authors:** Li Ping Wong

**Affiliations:** 1 Centre of Population Health (CePH), Department of Social and Preventive Medicine, University of Malaya, Kuala Lumpur, Malaysia; 2 Julius Centre University of Malaya (JCUM), Department of Social and Preventive Medicine, Faculty of Medicine, University of Malaya, Kuala Lumpur, Malaysia; University of Washington, United States of America

## Abstract

**Background:**

This study was a qualitative investigation into sexual attitudes and behaviours, and contraceptive use among Malaysian youth, based on constructs from the health belief model, theory of reasoned action, and problem behaviour theory.

**Methods:**

A total of 34 focus group discussions with 185 participants were conducted among the Malay (35%), Chinese (34%), and Indian (31%) young females between November, 2010 and April, 2011. The participants were secondary school students and university undergraduates from Selangor and the Federal Territory of Kuala Lumpur.

**Results:**

The study found a lack of knowledge about sexual issues and contraception among the participants. Many engaged in unprotected sexual intercourse and relied on periodic abstinence, natural methods, and traditional folk pregnancy preventive practices. The findings also revealed numerous categories of factors influencing sexual attitudes and behaviours: ethnic group and religion, level of religiosity, peer pressure and norms, and parental monitoring. With regard to condom use, factors such as embarrassment about condom acquisition, low perceived susceptibility to sexually transmitted infections (STIs), and perceived efficacy of traditional and folk methods of contraception, were uncovered from the discussions.

**Conclusion:**

This study underscores the importance of development of culturally specific interventions that address the identified promoting factors of premarital sex. Behavioral interventions to promote condom use should increase awareness about condom effectiveness against not only unwanted pregnancies but also STIs.

## Introduction

Although premarital sexual behaviour has been a critically important area of research and the literature concerning this issue is abundant, the understanding of premarital sexual behaviour in Eastern countries, particularly Islamic societies, is relatively scarce as sexuality remains a sensitive issue for many Muslims. Urbanization, modernization, and exposure to Western culture appear to have led to the erosion of traditional beliefs and values and the importance of virginity on marriage, and contributed towards more permissive attitudes towards sex [1]. Malaysia is a Muslim-majority country, with the majority being Muslim Malays and two other large ethnic groups, namely the Chinese and Indians. Malaysia is, in some respects, a socially conservative country with regard to reproduction and sexuality. Although being a moderate Muslim-majority nation, sex and related issues are sensitive and considered taboo for many in the three main ethnic groups.

In the past, there was no formal sex education in schools in Malaysia. The Malaysian government decided to introduce sex education in the schools beginning January 2011. Little is known about the sexual attitudes and behaviours of young unmarried women in Malaysia. Although there were national reports regarding adolescent sexual reproductive health knowledge and attitudes [2], [3], empirical evidence or published research in academic journals related to this issue is relatively scarce. A national study, conducted in 1995 by the National Population and Family Development Board (NPFDB) surveying Malaysian households, found that about 1% (13 of 1379) adolescents admitted that they had engaged in sexual intercourse and that most sexual encounters were unsafe, with no protection against sexually transmitted infections (STIs) and unwanted pregnancy [3]. A cross-sectional study in 2001 showed that 5.4% of a total of 4500 students, aged between 12 and 19 years, reported having had sexual intercourse [4]. A more recent nationwide survey, conducted in 2005, found that nearly 1.3% (25 of 1901) of young people aged between 15 and 24 years who were unmarried reported being sexually active [2]. It should be noted that these were self-report studies, and the extent of under-reporting of sexual conduct in sexual behaviour surveys has been reported to be high [5], therefore, the actual figure may be likely to be several-fold higher.

Premarital pregnancies place an economic burden on the young people involved and impact on society at large [6]. In Malaysia, this is evident in the increasing number of abandoned baby cases over the years. A Malaysia news media, The Star, 19 August 2010, reported that according to police reports, there were 65 baby abundaments throughout Malaysia in 2010. From 2005 to 2010, 472 babies were abandoned throughout the country, of which, 258 were dead and 214 were still alive. From the Islamic perspective, premarital sexual relationships are forbidden and persons committing the offence of *zina* (sexual intercourse without being validly married to each other) will be punished. Given the familial and social stigma attached to premarital sex, and the fear of punishment for the offence of *zina*, many resort to abandoning their newborn babies. Despite this trend, relatively few studies have explored in detail, in the Malaysian context, the sexual attitudes of young women who have never married and their behaviour. In the Malaysian context, understanding sexual attitudes, behaviour and contraceptive use is, therefore, critical in addressing the rising trend of premarital sexual intercourse and unwanted pregnancies from premarital sex. Such information can be used in designing evidence-based behavioural prevention-oriented interventions aimed at reducing the rate of sexual activity among the young and risky sexual behaviours.

Various theoretical models have been used to explain adolescent sexual attitudes and behaviours worldwide. An extensive survey of literature shows that the most commonly used theoretical frameworks for understanding adolescent sexual attitudes and behaviour patterns were the Health Behavioural Model (HBM), Theory of Reason Action (TRA) and Problem Behaviour Theory (PBT).

In the HBM, the core assumption is that individuals will take a specific health-related action if they have a positive expectation of avoiding the negative health condition. The HBM focuses on four perceptions that serve as the main constructs: 1) perceived susceptibility, belief in the chances of acquiring a condition; 2) perceived severity, belief in the seriousness of the condition; 3) perceived benefits, belief in the efficacy of the preventive behaviours that may prevent harmful conditions; 4) perceived barriers, belief in the tangible and psychological costs to one’s self from engaging in the action [7]. To help HBM better fit the challenges of the changing habitual unhealthy behaviours, the most recent addition to the HBM is the concept of *self-efficacy*, which is the perceived ability to successfully perform an action [8]. The HBM has generally focused on knowledge, and individual socio-demographic and cultural factors, such as the influence of family, peers and institutions (education and religion [9], and many studies have pointed out the links between sexual behaviour and these factors [10]–[12]. The HBM has also been used to understand contraceptive use. Women who perceived themselves at a greater perceived susceptibility to pregnancy were more likely to have used effective methods of contraceptive [13].

The TRA is based on the assumption that a person’s intention to perform a specific behaviour is a function of: 1) attitude towards the behaviour; and 2) the influence of the social environment on the behaviour [14]. TRA has been used in several studies to predict sexual behaviour and intentions. In particular, the TRA postulates that peers and parents have an influence in shaping individuals’ sexual attitudes and behaviours [15]–[17].

The PBT consists of three independent but related systems of psychosocial components: 1) the perceived environmental system, 2) the personality system, and 3) the behavioural system. The perceived environmental system consists of proximal and distal social influence factors, such as family and peer orientation, and expectations regarding problem behaviours. The personality system includes social cognitions, individual values, expectations, beliefs, and attitudes [18]. Social cognition features particularly prominent in PBT are parents, school, and peers, and, on the basis of the adolescent sexual initiation model, early sexual intentions were negatively associated with good parental communication and negatively associated with deviant peer affiliation [19]. PBT has been shown to be useful in predicting adolescent sexual behaviour and intentions [19], [20].

Given the scarcity of published data on sexual attitudes and behaviour among young Malaysians, the aim of this present study was to collect preliminary data on young Malaysians’ attitudes and provide descriptions of their experiences with sexually related issues using qualitative approach. We took full advantage of our ethnic diversity and explored the different ways in which religion and culture affect sexual attitudes and behaviour. The HBM, PTB and TRA served as the framework for discussions in this study. The use of multiple theories enables integration of the salient components of the theories to allow a more holistic explanation of behaviors [21]. Open-ended questions were posed to stimulate discussions address the different dimensions from the HBM, PTB and TRA, that served as the guiding framework of this study. Attitudes and behaviours relating to dating, courtship, and premarital sexual intercourse were assessed using the PBT and TRA models as a guiding framework. Specifically, the subjective norms of engaging in risky sexual behaviour (social or peer pressure, parental factors) with intent to undertake risky behaviour were probed in the discussions. Contraceptive attitudes and intention to use condoms associate the constructs of the HBM; perceived susceptibility, perceived severity, perceived benefits, and self-efficacy. The discussions were guided by the three models because they may be parts of a whole and complement each other in functional behavior.

This study differs from previous studies by exploring the perspective from a Muslim-majority country, coupled with its unique multicultural population of Malay (50.8%), Indian (6.9%) and Chinese (23.0%) origins (the aborigine and other minority groups making up to 11.0% and 8.3% of the population, respectively), the three largest ethnic groups in South East Asia. With increasing ethnic and cultural diversity across many countries around the world, the information from this study might potentially be of interest to many countries. Ultimately, the findings from this qualitative study may also be useful in identifying the best methodological designs and variables for future quantitative studies.

## Methods

Focus group discussions (FGDs) were conducted among two different groups of female participants: 1) secondary school students, 2) university undergraduates. In each group, discussions were stratified by ethnicity (Malay, Chinese, Indian) and dating status (have dated or currently dating someone, and never dated) in order to explore culturally diverse views on sexual attitudes and experiences. Because the primary intent of this study was to collect information on sexual attitudes and behaviours, the have dated or currently dating groups were further segregated into had or never had sexual relationship groups, in order to encourage open discussion and increase participants’ comfort levels.

The secondary school students were chosen from various community settings from Selangor and the Federal Territory of Kuala Lumpur, whereas the university women were from a public institution of higher learning in the Federal Territory of Kuala Lumpur. Secondary school students were considered for participation if they were of the designated ethnicity, between Forms 3 and 6, and currently living within the targeted postal codes. Likewise, the criteria for inclusion of university undergraduates were of the designated ethnicity and undergraduate students in their first to final years of the designated university. For both groups, participants were first recruited based on convenient sampling. A snowball technique was used to recruit participants who had had sexual experiences. In order to obtain a diversity of perspectives, opinions, and experiences, the FGD participants were asked to refer friends with both similar and different characteristics.

Most of the FGDs with university students were undertaken in quiet private rooms within the campus to ensure participants’ privacy and confidentiality. Several FGDs with secondary school students were conducted in the homes of the participants at their convenience, without their parents or guardians being present. The groups ranged in size from five to eight participants per session. The duration of each discussion generally lasted between 45 and 60 minutes. FGDs were moderated by a moderator and assisted by a note-taker experienced in focus group interview note-taking. Moderators and note takers were trained by the author on all aspect of FGD moderation. The moderator was trained to facilitate open and uninhibited discussions follow the FGD guide, posed questions from all study domains, and suggested probing questions. The note taker was trained in how to be objective in recording discussions and observing non-verbal expressions. Both the moderator and note-taker were female because of the sensitivities surrounding sexually related topics in the Asian culture. To keep the research focused on the main themes of the study, the moderator was given an FGD guide. The FGDs were conducted in English or in Bahasa Malay, the national language of Malaysia, which is widely spoken and understood.

### Focus Group Guide

The FGD guide consisted of four parts. The first part explored participants’ knowledge about sexual and reproductive health, which includes fertile period, pregnancy, and contraception. The second part accessed participants’ attitudes and behaviours relating to dating, courtship, and premarital sexual intercourse. In each focus group, the focus group moderator first guided the groups through conversation that is centered on cultural beliefs and norms attitudes about dating sexual related matters. Social and peer pressure to be sexually active, and parental control and monitoring as protective factor for sexual initiation were probed based on PBT and TRA constructs. The third part assessed participants’ attitudes and practices related to contraceptive use. Constructs arising from the HBM (perceived susceptibility, perceived severity, perceived benefits, and self-efficacy) were probed as factors associated with contraceptive use and nonuse, The last part of the guide consists of questions about information sources and communication related to sexual reproductive health.

The draft guide was pilot-tested in mock focus groups, in each of the secondary and university student groups, to ensure clarity in the order, timing, and wording of questions. The final version of the guide, which incorporated revisions based on the pilot-test findings, was used in subsequent focus groups. At least two research team members were present in each focus group; one member moderated the discussion, the other took detailed handwritten notes. All interviews were audio-recorded and transcribed thereafter. The numbers of FGDs were not predetermined but discussions were conducted until theoretical saturation had occurred and no new information was elicited.

Upon completion of the discussions, participants were asked to complete a demographic information sheet. As an attempt to capture the comprehensive conceptual structure of the behavioural theories, several close-ended questions, which elicit information about sexual intimacy, were also asked. The quantification results are illustrated in Table 1.

**Table 1:** Characteristic differences in secondary school (n = 83) and university female participants (n = 102).

Characteristic	Secondary school female n (%)	University female n (%)
***Personal background***		
**Age, years**		
Mean age (SD)	17.39(2.11)	22.06(1.69)
Range	14–19	19–26
**Ethnicity**		
Malay	30(36.1)	34(33.3)
Chinese	28(33.7)	34(33.3)
Indian	25(30.1)	32(31.4)
Others	**-**	2(2.0)
**Religiosity**		
Very religious	10(12.0)	19(18.6)
Somewhat religious	65(78.3)	71(69.6)
Not at all religious	8(9.6)	12(11.8)
***Family background***		
**Average household monthly income** a		
>MYR4000	6(7.2)	21(20.6)
MYR1000–4000	59(71.1)	66(64.7)
<MYR1000	18(21.7)	15(14.7)
**Parent’s discipline**		
Permissive	4(4.8)	2(2.0)
Moderate	57(68.7)	66(64.7)
Strict	22(26.5)	34(33.3)
**Locality**		
Urban	36(43.4)	61(59.8)
Suburban	4(4.8)	22(21.6)
Rural	43(51.8)	19(18.6)
***Sexual intimacy***		
**Dating status**		
Currently dating	37(44.6)	51(50.0)
Ever dated	21(25.3)	25(24.5)
Never dated	25(30.1)	26(25.5)
**Non-sexual physical affecting during dating** b, c		
Holding hands	50(60.2)	62(60.8)
Kissing on face	36(43.4)	46(45.1)
Kissing on lips	32(38.6)	42(41.2)
Hugging	38(45.8)	46(45.1)
**Ever had sexual intercourse**		
Yes	25(30.1)	37(36.3)
**Age of first sexual intercourse, years (SD)**		
Mean (SD) d	17.32(0.99)	19.84(2.30)

a 1 US Dollar = 3.0 Malaysian Ringgit (MYR).

b Responded by participants who have ever had dating relationship.

c Number of respondents do not sum up to 185 due to multiple-responses.

d Responded by participants who have ever had sexual intercourse.

### Data Analysis

The sampling process, data collection and analysis were continuous and iterative. All group discussions were immediately analysed and compared with the analysis of the previous discussions, which, in turn, further shaped the subsequent sampling, data collection and analysis. The FGDs were conducted until thematic saturation was reached or no new information was uncovered.

After transcription and cleansing, the transcripts were converted to rich text format and imported into NVivo software (QSR International Pty Ltd, Doncaster, Victoria, Australia) for coding and categorizing [22]. After several readings, key categories and themes from the participants’ narratives were developed. The codes were analysed using an interpretive descriptive method, where interpretative description goes beyond mere description and aims to provide an in-depth conceptual understanding of a phenomenon [23]. Coding was performed by a single coder and the consistency of coding was assessed by intra-coder reliability. The researcher coded segments of the data at two different periods and the intra-coder reliability was calculated as number of agreements divide by total number of agreements and disagreements. The calculated intra-rater agreement was in the 90th percentile range. Finally, the data were interpreted and presented using the respondents’ own words as illustrations.

### Ethical Considerations

The study was approved by the Medical Ethics Committee, University Malaya Medical Center, Kuala Lumpur, Malaysia. Due care was taken to ensure that all those who agreed to participate in the study did so voluntarily. Written informed consent was obtained from all participants prior to group discussions. For participants under 18 years of age, informed written consent was also sought from a parent or legal guardian.

The moderators explained the objectives of the study to all participants. Participants were informed that any information collected was to be kept confidential. No identifying information was obtained from any of the participants. Researchers were mindful of the sensitivity of the topics discussed and ensured that the research was undertaken in such a way as to establish a warm, empathetic relationship with the participants, thereby encouraging them to converse openly. We were careful to maintain confidentiality and show respect towards participants’ responses. Participants were not coerced to reveal their risky sexual practices. In an attempt to help participants feel at ease, in most of the discussions, the moderator would first guide the discussion surrounding perceptions of peer norms before specifically querying the participants’ own perspectives or practices.

## Results

A total of 34 FGDs (16 FGDs for secondary school students and 18 FGDs for university undergraduates) were conducted between November, 2010 and April, 2011. As shown in Table 1, the mean age was 17.39 (SD±2.11) and 22.06 (SD ±1.69) years, respectively. There were almost equal ethnic representations in both the secondary school and university undergraduate groups. The average household monthly income was MYR1,856.3 and MYR2,643.6 for the secondary school and university undergraduate groups, respectively, with most participants (n = 132, 71%) reporting an average household monthly income between MYR1,000 and MYR4,000. As a reference point, the Malaysian average monthly household income in 2009 was MYR4,025. Almost half of the participants in both groups noted that they were currently in dating relationships. Holding hands and kissing were common, and nearly one-third had had sexual intercourse. The mean age for sexual intercourse among the secondary school students in this study sample was 17.32(SD ±0.99) years.

### Sexual and Reproductive Knowledge

Overall, the participants in both groups had some knowledge of pregnancy, although considerable uncertainty existed as to the fertile phase of the woman’s menstrual cycle. Most of the participants knew that pregnancy can occur following vulval coitus. However, near half were unsure of the exact time of ovulation or that coitus should be avoided during this period to avoid conception. Additionally, some were not aware that the first incidence sexual intercourse could result in pregnancy. A greater proportion of secondary students than university undergraduates reported these knowledge deficiencies. Although participants who were currently dating had better sexual reproductive knowledge, a considerable number of participants who were currently dating lacked knowledge of fertile period and contraception.

*I know of it (fertile period), but I don’t know how to count (calculate their fertile days).* Malay, university undergraduate, dating, aged 24.

*I know there is a peak period but I don’t know the exact time.* Chinese, university undergraduate, dating, aged 25.

With regard to awareness of contraceptive methods, overall, most of the subjects had heard of some modern contraceptive methods. Condoms were the most widely known method, and nearly every participant across all FGDs mentioned them. Birth control pills were the next most commonly named method. The findings also show that a considerable number of participants were completely unaware of emergency contraceptive pills, including those in dating relationships, although the theme *contraceptive pills* or *birth control pills* was widely expressed by the participants. In the university undergraduate FGDs, likewise, emergency contraception remains widely unknown, even among the currently dating participants. Only a handful of participants across all the university undergraduate focus groups, of whom the majority were sexually active, knew about emergency contraceptive pills. Even among those that had heard about contraceptive pills as a method of preventing pregnancy, further probing revealed that most of the participants, including those who were sexually active, did know how to correctly take contraceptive pills. Subsequent probing by the moderator revealed that most had never thought of using contraceptive pills due to their preference and reliance on traditional or natural methods of contraception, and therefore had limited knowledge. When probed further, the participants stated that there were no religious prohibitions against using contraceptives outside marriage.

It was of interest that a considerable number of participants spontaneously mentioned unconventional techniques of contraception, rather than the use of modern methods of contraception, when queried about effective methods of pregnancy prevention. Additional probing revealed differences and similarities among the ethnic groups in response to types of unconventional contraception. The Malay participants cited ‘akar kayu’ (meaning root from tree) and ‘ubat periuk’ (*ubat* meaning medicine and *periuk* meaning cooking pot) which have long been used to avoid pregnancy. Only one participant, of Malay ethnicity, mentioned having heard about drinking ‘air jampi’ (water that had been subjected to incantation) as effective in preventing pregnancy. Besides herbal medicine, young fruit, namely pineapple, was commonly cited and emerged across a majority of the discussion groups. The Indian groups were more likely to cite papaya along with pineapple. Nevertheless, several Chinese participants pointed out that consuming large amounts of acidic fruits, such as lime and lemon, may increase the chance of miscarriage. Further probing revealed that these young fruits were believed to be ‘corrosive’ or ‘sharp’ and thus prevent conception. Some sexually active participants mentioned the effectiveness of drinking soda, or carbonated drinks, such as coke, in addition to young fruit juice. Likewise, coke was also regarded as a ‘sharp’ drink.

*Papaya and pineapple. My grandmother…she told my mum and my mum explained to me.* Indian, secondary school, dating, aged 18.

*My mum and I watched some movies, Indian movies. They mentioned it… if you eat papaya, and you eat pineapple. Pineapples kills… you know.* Indian, secondary school, dating, aged 17.

A considerable number of participants believed that jumping up and down after sexual intercourse and strong physical impact, such as falling down, may prevent pregnancy. This was generally agreed across most of the group discussions. Overall, focus groups revealed a higher knowledge about contraceptives among the dating than the never dated groups. Specifically, with regard to contraceptive types and usage, sexually experienced women were more knowledgeable than those who had no sexual experience.

### Dating and Premarital Sexual Intercourse

While the great majority of secondary school students across the three ethnic groups reported dating to be common among girls of their age, there were mixed perceptions about dating across the group discussions. With regard to the appropriateness of dating, some of them viewed dating and courtship at secondary school level as unsuitable as it may result in poor school performance and an increase in premarital sexual conduct. Among the university undergraduates, dating and courtship were more commonly reported, and many had no objections from their parents. For both groups, the majority of those in dating relationships were seeking emotional support, comfort and care, shared concerns and helped each other in their homework and studies. A participant explained: ‘*Because we need someone to care about us. When you find someone that you feel comfortable with…why not? I think this is very normal…for couples all over…very normal.*’ Chinese, secondary school, currently dating, aged18.

While the view of the great majority of participants across the three ethnic groups was that there are no religious and cultural prohibitions to dating and courtship, discrepancies in dating behaviours were observed among the ethnic groups. Many Malay participants stated that being in secluded places or partaking in physical intimacies, such as kissing, hugging and affectionate touching, are not permitted in the Islamic context. As a result, a few participants reported that they would often engage in group dating, where several couples would go out together. There were no mentions of religious prohibitions to physical intimacy during dating and courtship by the Chinese and Indian participants. Overall, less liberal attitudes towards dating intimacy were observed across the Indian groups. It was further revealed in the discussions that the attitudes towards dating intimacy of the Indian participants were largely shaped by strict parental supervision and parental attitudes. As the interviews proceeded, it was found that parental attitudes were also an important factor across other ethnic groups. This was evident in the relatively more conservative dating intimacy among participants who had strict parents than among those with liberal parents.

With regard to premarital sex, comparison across the three ethnic groups revealed that the Malay and Indian participants were more likely to indicate the importance of both religious and cultural values in attitudes towards premarital sexual permissiveness. Conversely, the Chinese participants were found to have more permissive premarital sexual attitudes throughout all the discussion groups. While the Muslim participants expressed religious prohibitions against premarital sex, the Indian participants were more likely to infer social and cultural prohibitions. Acculturation and degree of religiosity were found to be parallel with the premarital sexual permissiveness attitudes among the Malay participants. In addition, Malay participants who had had a sexual relationship and remained sexually active were more likely to rate their religiosity level as *Not at all religious* or *Somewhat religious* and to have come from an urban setting.

Narratives about their first experiences of sexual intercourse by secondary school students, especially those under 16 years of age, indicated that most of their sexual experiences were unexpected, unplanned or described as ‘just happened’. In contrast, it is more likely that the first incidence of intercourse among the university undergraduates had been a conscious decision as indicated in the following quote.

*Peer pressure or maybe curiosity also…because when one person (friend) is having it then you go like, ‘why is she having so much fun…? What is…in this thing that I’m missing out on?’…so you try it the first time and you go like…‘okay, it’s not that bad’…and then you just continue.* Indian, university undergraduate, currently dating, aged 23.

When parental attitudes towards premarital sexual permissiveness were probed, almost all participants, from both the secondary school student and university undergraduate groups, reported that their parents held conservative sexual attitudes and exhibited low permissiveness regarding premarital sex. Some participants reported that they restrained from sexual conduct because both their and their partners’ parents had been introduced and knew each other and forbade premarital sexual relationships. Additionally, there were fewer opportunities for sexual activity among the participants who were living with their parents. For this reason, secondary school students, who had initiated sexual intercourse and were living with their parents, reported engaging in intermittent rather than the regular intercourse of the university undergraduates, who were living away from home.

Another key theme that emerged in the results of the discussions was the importance of close parental monitoring on the child’s dating and sexual initiation. Although most of the participants who were in dating relationships or who had had sexual initiations reported lower levels of strictness in their parents’ discipline, the minority of the sexually active participants stemmed from strict parenting. Across all ethnic groups, many reported that their parents were unaware that they had already started having sex.

### Attitudes to and Practices of Contraception

The participants who did not use contraception for their first experiences of sexual intercourse cited that intercourse was unplanned. Although many reported consistent use of contraception in most experiences of sexual intercourse, a considerable number did not use any form of contraception. The focus groups revealed a great range of reasons for not practicing contraception. With regard to condom use, only one participant reported having partner who refused to use condoms. Non-use of condoms was not mainly due to their partners’ refusals but rather the participants themselves were of the opinion that condoms make sex less pleasurable. Further investigation revealed that, with regard to condom use, those who viewed themselves as somewhat religious did not differ from those who perceived themselves as not at all religious. For most participants, oral contraceptives were not preferred because of the perceived side effects as indicated in the following quote.

*“We should not use medication (contraceptive pills), because of side effects. The pills will damage our reproductive organ.”* Malay, secondary school, dating, aged 18.

Another key theme that emerged from the results of the FGDs was the reliance on unconventional pregnancy prevention approaches. When asked to name effective methods of pregnancy prevention, participants often mentioned unconventional contraceptive methods. Withdrawal and the safe period methods were commonly used by all sexually active participants. In the university graduate groups, some cited that post-coital douching may prevent pregnancy. The vast majority across all ethnic groups also revealed that, after intercourse, they drank plenty of fruit juices, commonly believed to prevent pregnancy, as indicated in the following quote.

*Sugarcane water, pineapple…yes, can prevent it. Let’s say you have intercourse, one week after that you just take pineapple juice and sugar cane and drink it. It is very strong for women, really it can clear it. You want to clean it, after one week or two weeks …take that.* Indian, university undergraduate, currently dating, aged 24.

Across the FGDs, structural barriers to obtaining contraceptives did not emerge as an important issue. Almost all cited that condoms are easily available at convenience stores when they need them. However, embarrassment in obtaining contraception was common, and was raised more frequently among the Malays than other ethnic groups. The majority reasoned that both social and religious disapproval of premarital sex affect access to contraception. Barriers to accessing contraceptives were also more commonly reported by the secondary school than the university undergraduate groups, and among those from rural rather than urban areas. The most common theme that emerged was related to the fear of being seen when obtaining contraceptives. The Chinese participants’ responses indicated that both social and religious factors have little influence on the acceptance and usage of any form of contraception.

Another key theme that emerged from the results of the FGDs was that contraception was used mainly to avoid unwanted pregnancy rather than the prevention of STIs. As a result, the main reason for not using contraceptives was the low perception of the risk of pregnancy. Many reported that they usually have intercourse during the infertile phase of the menstrual cycle. Inconsistent contraceptive use was also reported, which they then resolve using traditional folk methods to avoid pregnancy. Subsequently, when probed about their concerns regarding contracting an STI, many perceive their susceptibility to STIs to be low because they only have one partner or they are not sexually promiscuous, and therefore perceived themselves not to be at high risk of contracting STIs.

*My current boyfriend has brought it up before, and so I just told him, because I am not used to having sex with condoms, no need. I have had sex with condoms and I don’t like it. I think it feels different. I am not the kind that goes for multiple partners and stuff like that, and I don’t sleep around. And I trust him.* Indian, university undergraduate, currently dating, aged 24.

*I guess I’m pretty sure that nothing is going to happen, either it’s just after my period or before.* Indian, university undergraduate, currently dating, aged 23.

The majority of participants held the opinion that induced abortion, though illegal, is common among unmarried people. In general, in the religious and ethical contexts, participants viewed an abortion in the early stages of pregnancy as more acceptable than those performed in the more advance stages of pregnancy. When they were asked which abortion methods they believed were commonly used for inducing abortion, the majority held the opinion that folk remedies for abortion were widely used. The unconventional methods, similar to the methods used for contraception, such as eating large amounts of papaya and pineapple in early pregnancy, were widely mentioned across the group discussions, with some participants reporting that the methods had been used successfully by their peers. The Malay participants were more likely to report having heard that plants or herbs induce abortion, and the insertion of sharp objects, such as pineapple leaves, or putting pressure on the abdominal area were among the most popular methods for unconventional abortions. A Malay participant reported that she knew of someone who had had an abortion through forceful abdominal massage by a traditional village healer.

### Information Sources and Communication

The majority of the FGD participants across the different ethnic groups reported that they were more likely to seek information about sex and relationships from their friends and online sources. Compared with participants who were in dating relationships, those who were not in dating relationships were more likely to seek information from their parents. Likewise, secondary school students were also more likely than university students to converse with their parents about sex and relationships. The most common theme that emerged was related to embarrassment and fear of their parents perceiving or knowing that they had become sexually active. In some cases, participants described their parents as being insufficiently open-minded to discuss sexual issues. Although they were least likely to seek information from their parents, a considerable number of participants expressed a strong desire to seek the opinions of their parents on general matters of women’s reproductive health, such as puberty, menstruation and child birth.

## Discussion

In general, the study revealed a variable lack of knowledge about sexual reproductive health among the study participants. Despite lack of reproductive knowledge, dating or courtship was common even among the secondary school students, and there was evidence that secondary school adolescents and university undergraduates have had premarital sexual contact. Study also found low rates of conventional contraceptive use during sexual intercourse. Participants would prefer to rely on unconventional modes, such as withdrawal and the rhythm methods, or traditional folk methods. Reasons they did not use condom were low perceived susceptibility to STI and perceived condom decrease sexual pleasure. Main reason for contraceptive use was to prevent unintended pregnancy rather than protection against STIs.

Firstly, finding with regard to knowledge showed that many secondary school students lacked accurate knowledge about various aspects of sex and pregnancy. Prior studies noted that unintentional pregnancies among the young were due to misinformation and ignorance of sexual issues [24]–[25]. It was found that girls in secondary schools bear the greatest risks of unexpected consequences from sexual activity as most engaged in sexual intercourse without possessing adequate information about human reproduction and contraception [26]. Such a knowledge deficit was commonly reported and regarded as a complicated issue in conservative societies. A study has ruled out that a society that strongly disapproved pre-marital sex may deter women reproductive knowledge seeking and access to health services [27]. Further, it has also been noted that social taboos surrounding sexual reproductive health need to be minimized as it may also pose as barriers to women seeking reproductive health information or services [28], [29]. Therefore, in these societies, there is a need to expand access to youth-friendly sexual reproductive health services to enable the young to seek information and care while maximizing their confidentiality and privacy [29].

As dating or courtship was evident for many secondary school students and has become a popular norm when they enter tertiary education, the sex education should equip young women with adequate sexual reproductive health knowledge before they start dating. Specifically, the educational messages should include information about the risks, responsibilities, outcomes, and impacts of sexual actions. In this study, parents were found to play a role in the management of adolescent behaviour. Parental supervision and monitoring during the dating and courtship period were shown to be associated with delaying sexual onset [30].

Our findings showed evidence that secondary school adolescents and university undergraduates have had sexual intercourse although premarital sexual contact is unacceptable in Malaysian society and illegitimate in Islamic law. Among the sample of participants in this study, the Malays were more likely to refer to religious prohibition of premarital sexual contact. Further, among the Malay participants, the findings suggest that religion acts as a protective factor, and, in contrast, acculturation toward modern lifestyle acts as a promoting factor for attitudes of premarital sexual permissiveness. Those who held religion to be very important were associated with more conservative attitudes regarding sex. The finding of the association between religion and sexual attitudes in this study is congruent with the HBM and PBT explaining adolescent sexual behaviour as influence of social and cultural consciousness, and likewise reported in the previous studies [31]–[32].

The impact of acculturated attitudes toward modern lifestyle on sexual attitudes in adolescents and the young has similarly been reported in other studies [33]–[34]. Based on this finding, faith-based organizations may help shape young people’s values and attitudes towards sex. Faith-based initiatives have been successful in reducing the impact of the HIV epidemic in sub-Saharan Africa by promoting changes in sexual behaviour [35]. Future research concerning religious beliefs and practices in determining sexual attitudes and behaviour in Malaysia context may provide insight as to how faith-based programs be designed to shape positive attitudes towards sexual practices.

In order of increasing degree of acculturated attitudes toward modern lifestyle, particularly when it comes to sexually related matters, the Chinese participants in this study were more acculturated, followed by the Indians and the Malays. Although there is no religious prohibition on premarital sex for the Indians and Chinese, the Indians reported higher levels of social and cultural barriers to premarital sex than the Chinese. These ethno-religious disparities were evident in shaping the young’s dating and sexual risk-taking behaviours in this study. Thus, in the Malaysian context, sex education should be culturally sensitive and consider the ethno-religious disparities to effectively cater for all ethnic groups. Previous studies have shown that culturally tailored intervention has resulted in intentions to delay sex, to use contraceptives and to reduce sexual risk behaviour [36]–[37].

Overall, our study implies that both parental monitoring and parental strictness were not associated with sexual attitudes and behaviour, regardless of ethnic group and other demographic characteristics. Peer influence on sexual initiation was also evident. As predicted from the PBT and TRA, the findings suggest that both peers and parents shape the young’s sexual attitudes and behaviours. This association is well-established and has been widely reported [38]–[39]. Prior research has shown that poor parental monitoring and poor parent–teen communication and interaction were also associated with having deviant peers and the influence of peers on risky sexual behaviour [39]–[40]. The intervention of parents or guardians to increase monitoring and communication about sexual risks has been recommended [38]. In our sexually-conservative society, parental training on interventions to improve parents’ communication skills on sex issues is essential [41]. Interventions that address peer norms and peer pressure may also be especially important [42].

The study also demonstrated the great vulnerability to STIs of young people who are dating, due to not practicing contraception. That some of the participants did not protect themselves during their first incidence of sexual intercourse is worrisome. With regard to embarrassment associated with acquiring contraception, a private and confidential setting in which those vulnerable to embarrassment is needed for the purpose of overcoming embarrassment in acquiring contraception. Notably, erroneous belief that the contraceptive pills can be harmful was the barrier to women's use of oral contraceptive. Additionally, there were those who did not intend to use condoms, but instead relied on unconventional modes, such as withdrawal and the rhythm methods, or traditional folk methods. To date, there is no data to support the efficacy and safety of these traditional folk methods and these methods offer no protection from STIs. Many, including those who are sexually active, have high levels of perceived efficacy on traditional and folk methods of contraception. Compared to the Indian and Chinese participants, the Malays illustrated more types of traditional folk methods of contraception. Until further studies are performed, it is unclear if the religious and cultural norms of silence regarding sexual practices result in the heavy reliance on traditional folk methods of contraception. Therefore, the sexual health education module should include accurately information about oral contraceptives and the potential health hazards of traditional or folk methods. As male partner’s refusal to condom use was also uncovered in this study, women should also be informed of their rights and educated about their safe sex bargaining power to insist on condom use.

Incorrect or inconsistent use of contraceptives has been widely reported as an important cause of unintended pregnancies. It has been shown that nearly half of all unintended pregnancies occur among contraceptive users and that 90 percent of these pregnancies result from inconsistent or incorrect use of a method rather than from method failure [43]. This study found that myths and misinformation about modern contraception were common and associated with inconsistent use or not practicing modern contraception. Many lack knowledge and have concerns or fears about using contraceptive pills. In particular, participants were unaware of emergency contraception and lacked specific knowledge of taking the contraceptive pill correctly. The apparent lack of knowledge and misconceptions may limit efforts to obtain contraception and to continue using it, thereby increasing the risk of unintended pregnancy. Conventional family planning services, designed for married women, should also target providing services to the young. Concern that giving contraceptive services may promote premarital sexual intercourse can be overcome by developing specific skills for counselling the young [26]. Given that abortion is illegal in Muslim countries like Malaysia, prevention of unwanted pregnancies must always be given the highest priority and all attempts should be made to eliminate the need for abortion. Methods for termination of pregnancy that held no scientific evidence for safety and efficacy, such as the insertion of sharp objects and forceful abdominal massage or using herbs to induce miscarriage, emerged in the focus groups and may expose young people to various forms of reproductive health consequences, including damage to their reproductive systems. There is, therefore, a great need for education, advice and guidance regarding the risks and harms of self-induced abortions or abortions carried out by unqualified providers.

The FGD findings also revealed that many do not always practice safe sex despite having good reproductive and contraceptive knowledge. This implies that better knowledge does not necessarily translate into effective contraceptive practice. Most importantly, the data revealed that non-use of condoms was also due to perceived intercourse less pleasurable with condom and lack of perceived susceptibility to STIs and pregnancy. For many, sexual intercourse took place mainly in the infertile phases of the menstrual cycle to avoid pregnancy. Many perceived low vulnerability to STIs and view their partners as trustworthy, which correspond to the perceived susceptibility construct of the HBM. Previous studies showed that adolescents’ perceived risks of STIs not only influence their intentions to use condoms, but are associated with abstaining from sexual intercourse [44]. The findings suggest that the design of communication messages to increase condom use should be guided by the perceived susceptibility construct of the HBM.

Studies from other countries showed that unintentional pregnancies among the young could also be due to lack of power to negotiate with their partners on whether contraceptives should be used [24]–[25]. In contrast, coercion and lack of power in negotiating contraceptive use were not raised in our FGDs. Instead, women in this study considered that condoms interfering with sexual pleasure was a reason for non-use. This underscores the need to enlighten young Malaysian women of the consequences, in terms of high susceptibility to STIs, of engaging in unprotected sex. The FGD findings also revealed that religion and religiosity have less impact with regard to the use of condoms and contraceptive pills, which suggests that faith groups may, perhaps, have a less important role in helping to shape young women’s values and attitudes to contraceptive use.

Finally, the FGDs also generated valuable information on how to educate and disseminate information to adolescents and the young in a conservative society. Apart from the information, education and communication strategies, that range from awareness seminars, workshops, lectures, exhibitions, and television programmes [45], and sex education at school level, the importance of parent–teen communication about sexual matters is now widely recognized [46]. In common with that study [46], we found that young participants showed a tendency of wanting to learn about sexual matters from their parents. A worrisome finding, however, was that sexually active older participants in this study were even less likely to communicate with parents because of the fear of their parents finding out that they had had sexual intercourse. Parent–teen communication and adolescent sexual behaviour have been linked to less risky sexual behaviour among teenagers [47]. Thus parent–teen sexual communication should commence when the children are at secondary school level.

In interpreting these results, there are certain limitations with the study design that might impact upon the conclusions drawn. First and foremost, the convenience sampling may be biased toward including individuals who are more comfortable talking about sexual matters. Besides other acknowledged limitations associated with the use of qualitative methodology [48], the strength of this study lies in its large number of focus group discussions conducted with nationally-representative samples to obtain themes from a wide spectrum of participants and to ensure data saturation. Of note, the participants recruitment problems encountered in this study were similar to those in other studies [27]–[28]. Nevertheless by careful consideration and protection of the rights of the participants, their interests, sensitivities and privacy, this study has successfully recruited young sexually active unmarried participants from Muslim-majority communities, for in-depth qualitative inquiry involving sensitive and personal information related to sexual matters.

### Conclusion

The study shows clear differences in sexual attitudes and behaviours in a broad demographic sample of young people. Though a causal relationship cannot be established, the focus group findings shed much needed light on how numerous categories of factors can deter sexual attitudes and behaviours: ethnic group and religion, level of religiosity, peer pressure and norms, and parental monitoring. This study unearthed some important barriers to condom or use, particularly around condom attitudes and access. These include lack of sexual reproductive knowledge, false beliefs about modern contraception, embarrassment about condom acquisition, low perceived susceptibility to STIs, perceived efficacy of traditional and folk methods of contraception and male partner’s refusal to use condoms.

On the basis of the key findings discussed above, the first and foremost call of this study is for a culturally tailored intervention to delay sexual initiation and promote contraceptive use among young women. This study identified factors associated with young women’s sexual behaviour and contraceptive use, and identified areas that health authorities should assess and target for intervention. This study also shows that some of the attitudes on sex related matters and contraceptive use were associated with the theory based models and therefore underscores the importance of using theory-based models for the development of interventions aimed at reducing the rate of sexual activity among the young and changing their sexual behaviour. Nevertheless, given the qualitative nature of the study, future quantitative research is warranted to explore these associations, seek causal determination, and generalizability of the findings.

As part of a long-term strategy, intervention should be curriculum-based and implemented at secondary school level. The findings also highlight the importance of educating parents to improve parent–teen communication about sex. The study results have both theoretical and empirical implications for future research. Finally, this study is also a form of shared experience that can be used to assist other similar studies.
